# Integrating Proteomic Analysis and Machine Learning to Predict Prostate Cancer Aggressiveness

**DOI:** 10.3390/stats7030053

**Published:** 2024-08-21

**Authors:** Sheila M. Valle Cortés, Jaileene Pérez Morales, Mariely Nieves Plaza, Darielys Maldonado, Swizel M. Tevenal Baez, Marc A. Negrón Blas, Cayetana Lazcano Etchebarne, José Feliciano, Gilberto Ruiz Deyá, Juan C. Santa Rosario, Pedro Santiago Cardona

**Affiliations:** 1 Ponce Research Institute, Ponce Health Sciences University, Biochemistry and Cancer Biology Divisions, Ponce, PR 00716, USA; 2 Knight Cancer Institute, Oregon Health & Science University, Oncological Sciences Division, Portland, OR 97239, USA; 3 Universidad Central del Caribe, Department of Medicine, Bayamón, PR 00960, USA; 4 Universidad Autónoma de Guadalajara, Department of Medicine, Zapopan 45129, Mexico; 5 Ponce Research Institute, Ponce Health Sciences University, Surgery Division, Ponce, PR 00716, USA; 6 CorePlus Servicios Clínicos y Patológicos, Carolina, PR 00983, USA

**Keywords:** prostate cancer, aggressive, E-cadherin, N-cadherin, β-catenin, retinoblastoma, phosphorylation, epithelial to mesenchymal transition (EMT), CART

## Abstract

Prostate cancer (PCa) poses a significant challenge because of the difficulty in identifying aggressive tumors, leading to overtreatment and missed personalized therapies. Although only 8% of cases progress beyond the prostate, the accurate prediction of aggressiveness remains crucial. Thus, this study focused on studying retinoblastoma phosphorylated at Serine 249 (Phospho-Rb S249), N-cadherin, β-catenin, and E-cadherin as biomarkers for identifying aggressive PCa using a logistic regression model and a classification and regression tree (CART). Using immunohistochemistry (IHC), we targeted the expression of these biomarkers in PCa tissues and correlated their expression with clinicopathological data of the tumor. The results showed a negative correlation between E-cadherin and β-catenin with aggressive tumor behavior, whereas Phospho-Rb S249 and N-cadherin positively correlated with increased tumor aggressiveness. Furthermore, patients were stratified based on Gleason scores and E-cadherin staining patterns to evaluate their capability for early identification of aggressive PCa. Our findings suggest that the classification tree is the most effective method for measuring the utility of these biomarkers in clinical practice, incorporating β-catenin, tumor grade, and Gleason grade as relevant determinants for identifying patients with Gleason scores ≥ 4 + 3. This study could potentially benefit patients with aggressive PCa by enabling early disease detection and closer monitoring.

## 1. Introduction

Prostate cancer (PCa) is one of the most common cancers, accounting for 288,300 estimated new cases in men in 2023, and is the second leading cause of cancer-related deaths in men in the United States [1]. This disease is characterized by rapidly dividing cells in the prostate gland, resulting in abnormal prostate gland growth [2]. Only 8% of all diagnosed PCa cases invade beyond the prostate, metastasizing and affecting men’s quality of life [3]. Unnecessary treatment may result in side effects such as gynecomastia, decreased libido, incontinence, and erectile dysfunction, among many others [4]. Thus, accurately predicting the aggressiveness of PCa can help avoid overtreatment, which occurs in 1.7–66% of cases, and tailor personalized treatments to each patient [5]. However, biologically predicting which form will be aggressive has always been a challenge [3,6]. Biomarker tests such as Prolaris and Oncotype DX have been developed to assess the risk of aggressive PCa [7]. For example, Oncotype Dx measures the genetic expression of cancer-related genes and is used to predict the probability of adverse pathology, cancer-related death, and metastasis within 10 years [8]. However, no prospective data confirm the superiority of active surveillance oven treatment based on this test [9]. Prolaris is another genomic test that measures the gene expression of cell cycle progression genes to predict disease progression in men with localized PCa [10]. Yet, a study on its utility found no significant impact on treatment plans or patient outcomes and identified insufficient data to inform cost-effectiveness [11]. Thus, there is a lack of test comparisons regarding their ability to predict aggressive PCa at initial or subsequent prostate biopsy [12]. Therefore, identifying new and reliable biomarkers is essential in accurately predicting aggressive PCa, to improve the current available methods.

In this study, we employed logistic regression models, classification trees, and regression trees to determine the likelihood of aggressive PCa. Logistic regression is a fundamental tool in cancer research for predicting the likelihood of events, facilitating tumor classification, prognosis, risk assessment, and response prediction [13]. This method assigns a coefficient to each evaluated variable in the model, indicating the extent to which each variable affects disease progression, making it suitable for clinicians to understand. In contrast, classification trees and regression trees are decision-tree-based models that differ in their approaches and applications. Regression trees are analytical tools that can explore complex relationships and are designed to predict categorical outcomes based on the most informative features provided to the model. Classification trees, however, are designed to predict a categorical outcome based on the most informative features provided to the model. In clinical settings, decision trees have strengths, compared to other classification techniques, because of their decision-making capability and ability to classify unknown datasets [14]. Both classification and regression tree (CART) models offer a different structure for determining the relationships between variables, allowing for a comparison with linear regression models to provide a better understanding of how to precisely identify aggressive PCa and ultimately improve patient outcomes.

Cadherins present themselves as promising candidates for identifying aggressive PCa, due to their established role in cell adhesion and metastasis. E-cadherin, a key member of the cadherin family, is involved in maintaining the integrity of adherens junctions and epithelial phenotype organization, in a complex with β-catenin [15]. In adults, the loss of E-cadherin at cell-to-cell contacts and its complex with β-catenin is associated with increased cell motility and advanced stages of cancer [16]. Alterations in the E-cadherin cell–cell adhesion mechanism appear to be present in almost half of all PCa cases and may contribute to the acquisition of metastasis in aggressive PCa [17]. Indeed, the functional loss of E-cadherin in epithelial cells is considered a hallmark of epithelial-to-mesenchymal transition (EMT), in which epithelial cells change into motile mesenchymal cells and express mesenchymal markers such as N-cadherin, leading to cancer spread and metastasis [18,19]. N-cadherin is typically absent or expressed at low levels in epithelial cells; thus, its expression in cancer cells is associated with tumor aggressiveness and disease progression [20,21]. The loss of E-cadherin and upregulation of N-cadherin during EMT is known as cadherin switching, which promotes the disassociation of E-cadherin with β-catenin and establishes a relatively weak adherens junctions with N-cadherin [22].

E-cadherin expression can be downregulated by the tumor suppressor retinoblastoma (Rb) protein [23]. The Rb protein is commonly known to be an important cell cycle regulator, and losing its tumor suppressive function allows unregulated cell cycle progression and promotes tumor growth [24]. Importantly, the Rb function is controlled by post-translational modifications, including phosphorylation. However, there is little evidence regarding the functional role of these Rb phosphorylation sites. The phosphorylation of Rb at Serine 249 (Phospho-Rb S249) is in the N-terminal domain of Rb, which is not yet fully understood, however, a previous lung cancer study in our lab identified that Phospho-Rb S249 levels positively correlated with a higher tumor grade [25]. Thus, EMT markers, such as N-cadherin, β-catenin, and E-cadherin expression, accompanied by the expression of phosphorylated Rb at S249 may be valuable biomarkers for predicting patients harboring aggressive PCa. In this study, we developed and evaluated logistic regression and CART models to assess Phospho-Rb S249, N-cadherin, β-catenin, and E-cadherin expression levels as potential biomarkers for identifying aggressive PCa. We hypothesized that the combined expression of these proteins could serve as biomarkers for identifying aggressive PCa, using logistic regression and CART models. The results revealed that the classification tree was the most effective method for identifying patients with aggressive PCa (Gleason scores ≥ 4 + 3) by incorporating β-catenin, tumor grade, and Gleason grade as important determinants. These findings might benefit patients with aggressive PCa, who may benefit from early treatment interventions and closer monitoring, potentially facilitating the management of aggressive PCa.

## 2. Materials and Methods

### 2.1. Tumor Microarrays (TMAs)

PCa TMAs were obtained from the company US Biomax (Derwood, MD, USA). Samples were formalin-fixed, paraffin-embedded, and mounted on a positively charged glass. Each tumor sample was 5 μm in thickness, and US Biomax provided the clinicopathologic data for each patient, consisting of the age, grade, stage, Gleason score, and Gleason grade. They also provided information regarding the tumor size, lymph nodes invasion, and metastasis (TNM). The TMA catalog numbers used were PR633, PR721, PR483d, PR484a, PR803d, PR807c, and PR1921c. The tissue samples analyzed in our study were commercially available tumor microarray (TMAs) acquired through the company US Biomax. No samples were excluded from the analysis unless the clinicopathologic data being analyzed were not provided by US Biomax.

### 2.2. Immunohistochemistry (IHC)

The process of IHC was performed to target the proteomic expression of Phospho-Rb S249, N-cadherin, β-catenin, and E-cadherin on the PCa TMAs following a previously published protocol [26]. Each patient was subjected to IHC with antibodies against the expression of Phospho-Rb S249, N-cadherin, β-catenin, and N-cadherin. The following antibodies were used: purified mouse anti-N-cadherin (BD Biosciences, Franklin Lakes, NJ, USA; Cat. No.610920) 1:125; β-catenin (Cell Signaling, Danvers, MA, USA; Cat. No. 8480S) 1:200; E-cadherin (Cell Signaling, Danvers, MA; Cat. No. 3195S) 1:400; and Phospho-Rb S249 (Abcam, Waltham, MA, USA; Cat. No. ab4788) 1:100. The secondary antibody used was the Super Sensitive Link Label IHC kit (BioGenex, Fremont, CA, USA; Cat. No. LP000-ULE). Image acquisition was performed using the Nikon NIS-Elements software AR 2.22.25. All tissue cores were analyzed in 4 quadrants at 40× visual fields to consider the whole tumor area and heterogeneity. Each immuno-stained TMA was independently and blindly scored for the number of immune-positive cells. The scoring was performed using two approaches: using the ImageJ program version 1.54d and three independent/blinded observers, to combine subjective and objective measures. Each of the three observers analyzed the individual tissues in 4 quadrants at 40× magnification per visual field per patient. The scoring system used by the observers ranged from 1 to 4, where 1 meant a negative result, and 2, 3, and 4 meant low positive, positive, and high positive, respectively. No data points were excluded from the analysis.

### 2.3. Statistical Analysis

To identify associations between variables, we employed Spearman’s rank correlation coefficients using GraphPad Prism software version 9. Spearman’s rank correlation coefficients were calculated to assess associations between non-normally distributed variables, as determined by a Shapiro–Wilk normality test. Further, patients were stratified by their Gleason scores (≤3 + 4 and ≥4 + 3), an architectural grading system ranging from 2 to 10; patients harboring Gleason scores ≤ 3 + 4 tend to have a better prognosis compared to those with Gleason scores ≥ 4 + 3 [27,28]. The Gleason scores (≤3 + 4 and ≥4 + 3) were analyzed as a dependent variable, whereas the clinicopathologic data (excluding the Gleason score) and biomarker expression were analyzed as independent variables. Additionally, patients were stratified according to their E-cadherin staining patterns (normal or aberrant) because previous studies have exploited the normal and aberrant E-cadherin staining patterns to identify cancer dissemination and predict patient survival [29,30]. The E-cadherin staining pattern was analyzed as a dependent variable and the clinicopathological data and biomarker expression (excluding E-cadherin) as independent variables. After stablishing these patients’ stratification, we performed the subsequent analyses. To compare biomarker expression within the Gleason scores and E-cadherin staining patterns, a non-parametric *t*-test was used, as each patient stratification group was an independent cohort. Subsequently, the data were subjected to logistic regression and classification and regression tree (CART) analyses to determine if clinicopathological data and biomarker expression (independent variables) can accurately distinguish between Gleason scores and E-cadherin staining patterns (dependent variables). We used different model comparison criteria to determine the performance of our models in distinguishing aggressive PCa. The performance metrics used were pseudo-R^2^ (logistic regression), area under the curve (AUC), and pseudo-R^2^ (classification tree), as well as R^2^ and root mean squared error (RMSE) (regression tree). The models were performed as detailed below.

#### 2.3.1. Logistic Regression

Two logistic regression models were generated using STATA 17.0, each accompanied by descriptive statistics. Clinicopathological data and biomarker expression were coded as binary variables for the models. The first logistic regression model stratified patients by their Gleason scores (≤3 + 4 and ≥4 + 3). The second logistic regression model stratified patients by their E-cadherin staining patterns (normal or aberrant). For both patient stratifications, descriptive statistics were generated for each variable, including the mean, standard deviation, median, and percentiles (e.g., 25th and 75th percentiles) for each biomarker expression and the clinicopathologic data. This model provided odds ratios (ORs) and confidence intervals (CIs) to quantify the strength and direction of the associations between independent and dependent variables in each model. Statistical significance was established at *p*-value < 0.05.

#### 2.3.2. Classification Tree

We generated the classification trees using R studio V. 4.3.0. We generated the models to predict the Gleason scores (first model) and E-cadherin staining patterns (second model). The dataset used for both models was randomly split into training and testing subsets in a 4:1 ratio and a minimum node size of 30 observations. The trained tree was subsequently pruned to optimize performance and prevent overfitting, using the optimal cp value, determined from the lowest cross-validated error. The pruned classification tree performance was evaluated by measuring the area under the curve (AUC) to assess predictive ability and discrimination capability. The pseudo-R-squared (pseudo-R^2^) values were also calculated to measure the model’s explanatory power. Additionally, a confusion matrix was generated to provide details of the classification performance, including metrics such as sensitivity, specificity, positive predictive value (PPV), negative predictive value (NPV), prevalence, detection rate, detection prevalence, and balanced accuracy.

#### 2.3.3. Regression Tree

The regression tree analysis was conducted using R studio V. 4.3.0 to model the relationship between the biomarker’s expression and the clinicopathological data with the Gleason scores (first model) and E-cadherin staining patterns (second model). The data were split into training and testing sets in a 4:1 ratio and a minimum split of 30 observations. The tree’s cp value was adjusted with the lowest cross-validated error to prune the tree. The pruned regression tree performance was evaluated using the root mean squared error (RMSE), which provides an indication of the average magnitude of the prediction errors, with lower values indicating better model performance. Additionally, to ensure a thorough evaluation, the R-squared (R^2^) value was assessed to measure the proportion of variance in the dependent variable (Gleason scores or E-cadherin staining patterns) explained by the model.

## 3. Results

### 3.1. Phospho-Rb S249 as Biomarker for the Identification of Aggressive PCa

**Figure 1.**
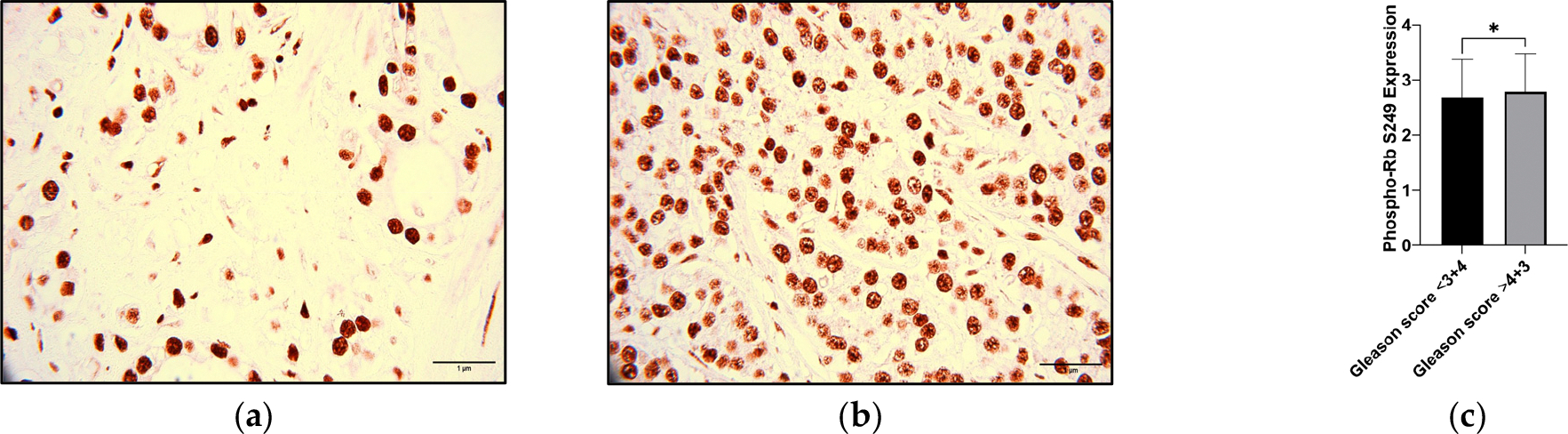
Phosphorylation of Rb at S249 (Phospho-Rb S249) expression in PCa tissue. This figure shows the immunohistochemistry (IHC) staining pattern of Phospho-Rb S249 in PCa tissue, with distinct Gleason scores. The images depict the staining pattern in patients with (**a**) Gleason score ≤ 3 + 4, a less aggressive form of PCa, and (**b**) Gleason score ≥ 4 + 3, a more aggressive form of PCa (**c**). A comparison of Phospho-Rb S249 expression between the two Gleason score classifications is shown. A non-parametric *t*-test was employed to assess the statistical significance of these findings; * *p* < 0.05. All tissue sections were analyzed in 40× visual fields, with representative images captured at 100× for better visualization (N = 403).

To validate the potential of the phosphorylation of Rb at S249 (Phospho-Rb S249) in identifying aggressive prostate cancer (PCa), we conducted the immunohistochemistry (IHC) of PCa tumor microarrays (TMAs). Figure 1 illustrates the expression of Phospho-Rb S249, which increases with increasing Gleason score. Patients with lower Gleason scores (Figure 1a) had lower Rb phosphorylation at S249; in contrast, patients with higher Gleason scores had higher Rb phosphorylation at S249 (Figure 1b). To compare the Phospho-Rb S249 expression within the Gleason scores ≤3 + 4 and ≥4 + 3, a non-parametric *t*-test was performed. Results confirmed that the Phospho-Rb S249 expression was higher within Gleason scores ≥ 4 + 3 (*p*-value: 0.0432), suggesting its potential for identifying men with aggressive PCa (Figure 1c). To assess the relationship between Phospho-Rb S249 expression and the clinicopathological data of the PCa patients, we conducted a correlation coefficient analysis (N = 403). The results showed that Phospho-Rb S249 positively correlated with tumor size (*p*-value: <0.0001, r: 0.4711) and stage (*p*-value: <0.0001, r: 0.4405) (Table 1), suggesting an association of Phospho-Rb S249 levels with the acquisition of aggressive PCa features.

### 3.2. N-Cadherin, β-Catenin, and E-Cadherin as Biomarkers for the Identification of Aggressive PCa

**Figure 2.**
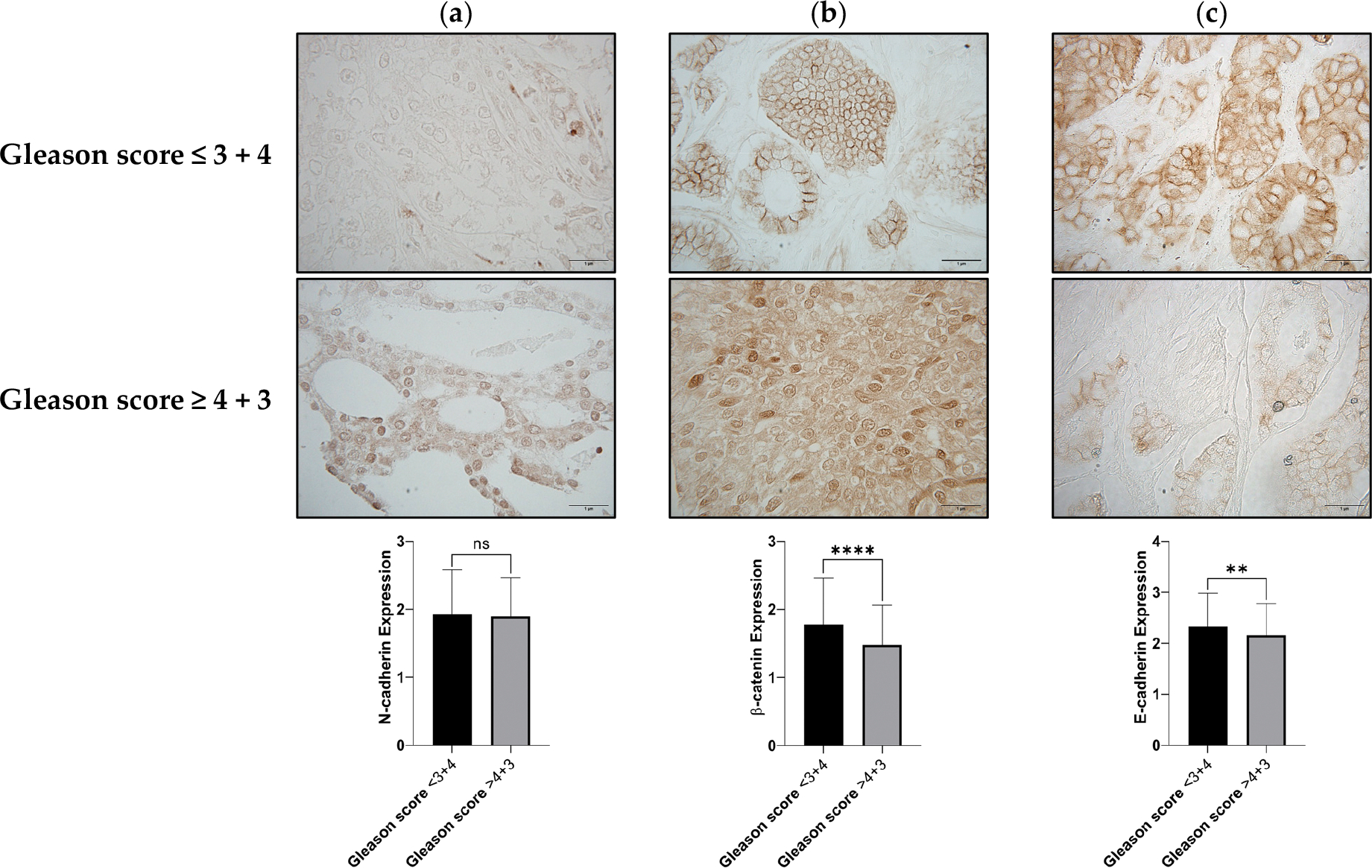
IHC staining of adhesion molecules in PCa tissue. This figure shows the IHC staining of N-cadherin, β-catenin, and E-cadherin in PCa tissue, with distinct Gleason scores (≤3 + 4 and ≥4 + 3). (**a**) N-cadherin, (**b**) β-catenin, (**c**) E-cadherin. The results presented in the upper section of the figure correspond to a patient with a Gleason score of 3 + 3, representing the staining patterns that predominate within the Gleason scores ≤ 3 + 4, a less aggressive form of PCa. In the lower section of the figure, the staining corresponds to a patient with a Gleason score of 5 + 4, representing the pattern that predominates in patients with Gleason scores ≥ 4 + 3, a more aggressive form of PCa. A non-parametric *t*-test was employed to assess the statistical significance of these findings; non-significant (ns), ** *p* < 0.01, and **** *p* ≤ 0.0001. All tissue sections were analyzed in 40× visual fields, with representative images captured at 100× for better visualization (N = 403).

To evaluate if N-cadherin, β-catenin, and E-cadherin could identify aggressive PCa, we evaluated their membranal expression by conducting the IHC of PCa TMAs. By specifically analyzing their membranal expression, in which they likely contribute to cell adhesion, we took into consideration the fact that these epithelial-to-mesenchymal (EMT) biomarkers may change their intracellular location when advancing to a more aggressive disease. Figure 2 shows the IHC staining from N-cadherin, β-catenin, and E-cadherin expression on the PCa TMAs (N = 403). The results showed that N-cadherin expression poses non-statistical differences when comparing its expression within different Gleason scores (Figure 2a). In addition, we found that β-catenin and E-cadherin expression reduces with increasing Gleason scores (Figure 2b,c), suggesting a potential cell-to-cell detachment, related to the development of aggressive PCa.

We also investigated the correlation between the expression of N-cadherin, β-catenin, and E-cadherin with the clinicopathologic data of the PCa patients (Table 2). The results showed that N-cadherin expression positively correlated with tumor size (*p*-value: <0.0001, r: 0.3782), stage (*p*-value: <0.0001, r: 0.3522), and Gleason grade (*p*-value: 0.0313, r: 0.1643). The β-catenin expression was found to negatively correlate with tumor size (*p*-value: 0.0119, r: −0.1861), grade (*p*-value: 0.0004, r: −0.2716), stage (*p*-value: 0.0051, r: −0.2069), Gleason grade (*p*-value: 0.0010, r: −0.2495), and Gleason score (*p*-value: 0.0002, r: −0.2810). The E-cadherin expression negatively correlated with tumor size (*p*-value: 0.0002, r: −0.2746), grade (*p*-value: <0.0001, r: −0.2984), stage (*p*-value: <0.0001, r: −0.3567), Gleason grade (*p*-value: 0.0020, r: −0.2338), and Gleason score (*p*-value: 0.0008, r: −0.2530). The combined findings may indicate that the reduced expression of β-catenin and E-cadherin, accompanied by the upregulation of N-cadherin expression, may be suggestive of weaker cell-to-cell connections between prostate cells and a shift towards a more aggressive phenotype.

### 3.3. Models for the Detection of Patients with Gleason Scores ≥ 4 + 3

#### 3.3.1. Logistic Regression

To predict the probability of having an aggressive PCa, we stratified the patients based on their Gleason scores (≤3 + 4 or ≥4 + 3), and we evaluated the distribution of the clinicopathologic data and the expression of biomarkers (Phospho-Rb S249, N-cadherin, β-catenin, and E-cadherin) (Tables 3 and 4) in each category through a bivariate analysis. This allowed us to identify differences in the distribution of these variables depending on whether the patients had a high or low Gleason score. Table 3 displays the clinicopathologic data of the patients within each Gleason score group. Statistically significant differences were obtained between the means of the tumor size (*p*-value: 0.0002), stage (*p*-value: 0.0018), metastasis (*p*-value: 0.0089), grade (*p*-value: 0.0000), and Gleason grade (*p*-value: 0.0000). Subsequently, we proceeded to assess the mean expression of the biomarkers Phospho-Rb S249, N-cadherin, β-catenin, and E-cadherin within each Gleason score (Table 4). We identified significant differences between the mean expression of Phospho-Rb S249 (*p*-value: 0.0304) and β-catenin (*p*-value: 0.0003). These results suggest that the combined expression of Phospho-Rb S249 and β-catenin may have the potential to predict the probability of having an aggressive PCa based on Gleason scores. Thus, the clinicopathologic data and biomarkers who showed differences in the bivariate analysis were selected to conduct a logistic regression model.

A logistic regression model was then performed to evaluate if these variables could predict patients with Gleason scores ≥ 4 + 3 (N = 396). The results showed a negative association of β-catenin (OR: 0.55) with the identification of Gleason scores ≥ 4 + 3 (Table 5). This means that for every one-unit increase in membranal β-catenin expression, the odds of having a PCa patient with a Gleason score ≥ 4 + 3 are reduced by 45%. Thus, having lower membranal β-catenin levels is associated with an increased chance of having aggressive PCa. The model performance was calculated by measuring the pseudo-R^2^, obtaining a value of 0.6108, which indicates that the model correctly classifies 61.08% of the instances, suggesting it has a good ability to classify patients by their Gleason scores.

In addition, we analyzed the individual biomarkers and explored different combinations of them to develop a logistic regression model, as detailed in Supplementary Tables S1–S3. Our analysis revealed that when examined separately, only β-catenin and E-cadherin showed potential to distinguish patients with Gleason scores ≥ 4 + 3 (Supplementary Table S1). However, combining biomarkers is crucial to accurately identify PCa patients based on their Gleason scores. Thus, when making different biomarker combinations, the results are consistent with those presented in Table 5, which indicates that β-catenin can distinguish patients with Gleason scores ≥ 4 + 3 (Supplementary Tables S2 and S3). This finding suggests that β-catenin is a key biomarker for distinguishing PCa patients with Gleason scores ≥ 4 + 3.

#### 3.3.2. Classification Tree

**Figure 3.**
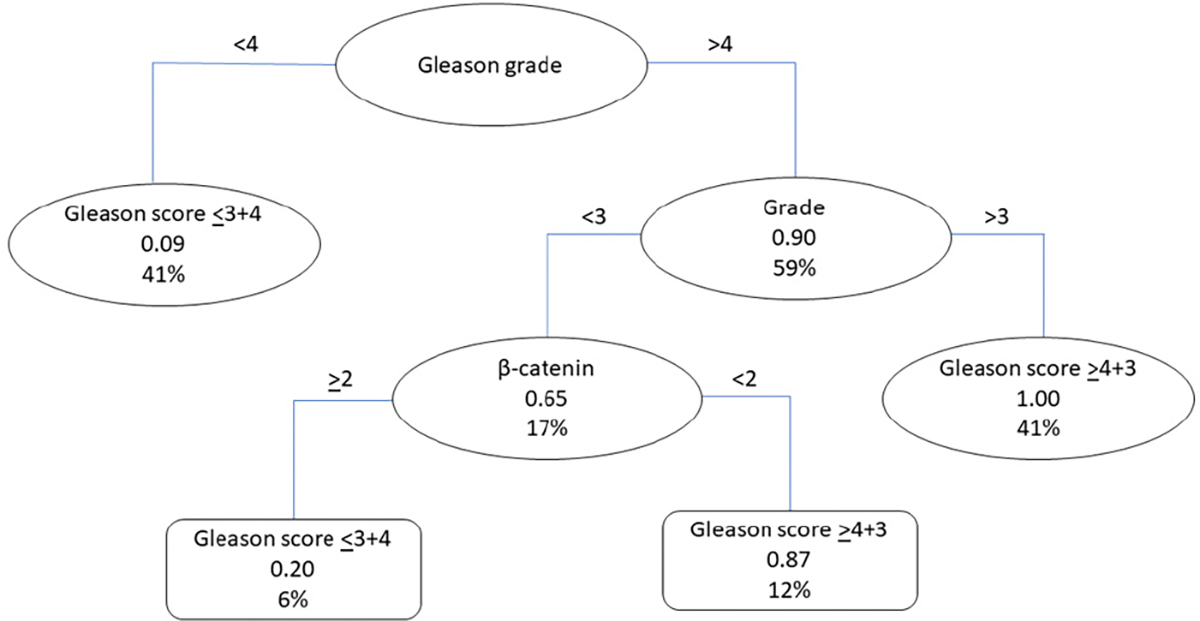
Classification tree for identifying PCa with Gleason scores ≥ 4 + 3 (N = 396).

We developed a classification tree to predict the probability of having a patient with a Gleason score ≤3 + 4 or ≥4 + 3. We considered the expression levels of biomarkers (Phospho-Rb S249, N-cadherin, β-catenin, and E-cadherin) and clinicopathological data as predictor variables. The proposed classification tree, comprising five nodes and two leaves, is shown in Figure 3. The significant variables identified by the model were β-catenin expression, the Gleason grade, and grade (Figure 3). The Gleason grade was the primary split criterion, with patients having a Gleason grade < 4 having a 0.09 (9%) probability of having a Gleason score ≤ 3 + 4. In contrast, patients with a Gleason grade of >4 were further distinguished by tumor grade. Patients with a tumor grade > 3 had a 1.00 (100%) probability of having a Gleason score > 4 + 3. In patients with a tumor grade < 3, the model considered the β-catenin expression levels. Specifically, patients with high β-catenin levels had a 0.20 (20%) probability of having a Gleason score ≤ 3 + 4. In contrast, patients with lower β-catenin levels had a 0.87 (87%) probability of having a Gleason score ≥ 4 + 3.

The performance of this classification tree was evaluated using several metrics to assess its ability to distinguish between Gleason scores (Table 6). The model achieved a high area under the curve (AUC) of 0.9572 for the training set and 0.9621 for the testing set, indicating a strong performance in distinguishing between Gleason scores. The pseudo-R^2^ values for the training and test sets were 0.7401 and 0.6295, respectively, indicating a good fit for both the training and testing data. This means that the model explains approximately 74% and 63% of the variance in the data, respectively. In terms of sensitivity and specificity, the model achieved high values of 0.9655 and 0.9133, respectively, indicating that it effectively identifies true positives (patients with Gleason scores ≥ 4 + 3) and true negatives (patients with Gleason scores ≤ 3 + 4). A positive predictive value (PPV) of 0.8960 suggests that when the model predicts a high Gleason score, there is a high probability that the prediction is correct. Conversely, the negative predictive value (NPV) of 0.9716 indicates that when the model predicts a low Gleason score, it is highly accurate. The balanced accuracy obtained was 0.9394, confirming the robustness of the model. Overall, these results indicate that the combined predictor variables of Gleason grade, grade, and β-catenin are highly effective tools for identifying patients with Gleason scores ≥ 4 + 3.

#### 3.3.3. Regression Tree

**Figure 4.**
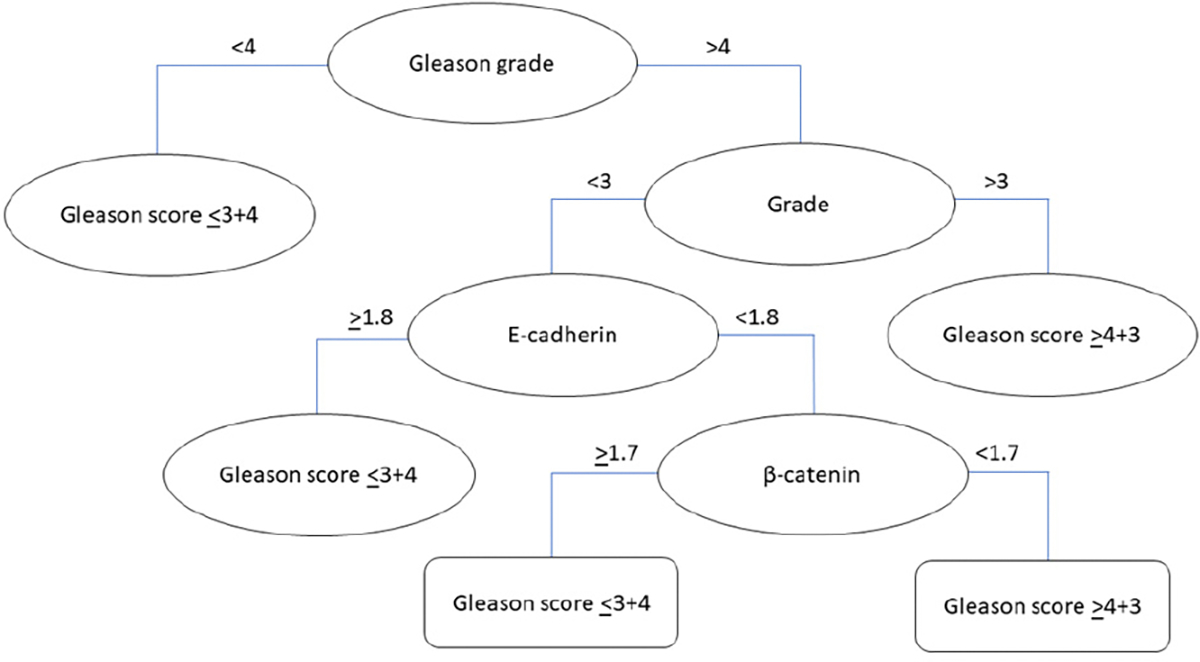
Regression tree for identifying PCa with Gleason scores ≥ 4 + 3 (N = 396).

The regression tree shown in Figure 4 consists of seven nodes and two leaves. This tree highlights the Gleason grade as a critical variable for identifying Gleason scores ≥ 4 + 3, setting this variable as a primary split criterion. The model predicts that patients with Gleason grades < 4 will have a Gleason score ≤ 3 + 4. However, for patients with a Gleason grade > 4, the model makes further distinctions based on the tumor grade. If the tumor grade was >3, the predicted outcome was a Gleason score ≥ 4 + 3. However, if the tumor grade was <3, patients were further distinguished by the expression levels of E-cadherin and β-catenin. The results showed that patients with high E-cadherin expression had a predicted outcome of a Gleason score ≤ 3 + 4, while those with lower E-cadherin levels were further split by β-catenin levels. If β-catenin levels are high, the prediction is a Gleason score ≤ 3 + 4, however, if β-catenin levels are lower, the predicted outcome is a Gleason score ≥ 3 + 4. To evaluate the performance of the model, we analyzed the R-squared (R^2^) and root mean squared error (RMSE) metrics (Table 7). An R^2^ value of 0.6961 indicates that approximately 69.62% of the variability in the Gleason score classifications (≤3 + 4 and ≥4 + 3) is explained by the model, suggesting a strong relationship between the predictors and the target variable. An RMSE of 1.0247 suggests that the model’s predictions are accurate, but there is some deviation from the actual Gleason scores, with predictions deviating by approximately one category level on average.

### 3.4. Models for the Detection of Patients with Aberrant E-Cadherin Staining Patterns

#### 3.4.1. E-Cadherin Staining Patterns

**Figure 5.**
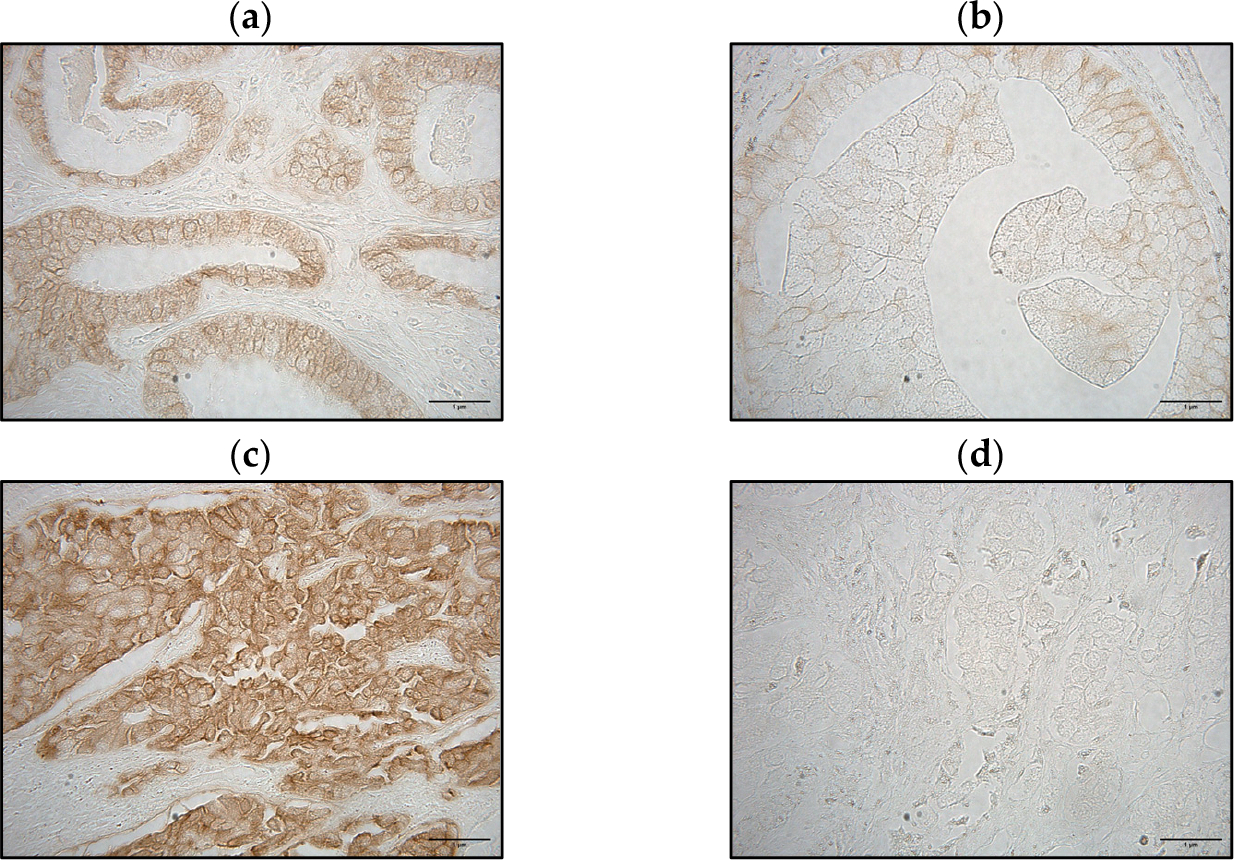
E-cadherin staining pattern on PCa tissue (**a**). Membrane staining classified as normal; located at cell–cell contacts (**b**). Aberrant staining classified as heterogeneous; some cells exhibit membrane staining and others exhibit a negative staining (**c**). Aberrant staining classified as cytoplasmic; cells exhibit abnormal cytoplasmic staining instead of membrane staining (**d**). Aberrant staining classified as a completely negative staining; none of the tumor cells are stained. All tissue sections were analyzed in 40× visual fields, with representative images captured at 100× for better visualization (N = 403).

E-cadherin is a well-established cell–cell adhesion molecule, essential for maintaining stable epithelial cell–cell contacts. Previous studies have highlighted that the normal and aberrant E-cadherin staining patterns helps identify cancer progression [31,32]. Thus, we classified the PCa patients according to the normal or aberrant E-cadherin staining patterns. The normal pattern, characterized by membrane-localized expression, was used to distinguish cell-to-cell attachment (Figure 5a). In contrast, the aberrant pattern exhibited several expression patterns, including heterogeneous, cytoplasmic, and negative staining. The heterogeneous pattern displayed some cells with membrane staining, whereas others did not exhibit any signal (Figure 5b). Additionally, we identified a pattern in which E-cadherin appeared within the cytoplasm (Figure 5c). Notably, we also observed a negative pattern, where no E-cadherin staining was detected in the PCa cells, indicating loss of E-cadherin expression (Figure 5d).

#### 3.4.2. Logistic Regression

After stratifying the PCa patients by their E-cadherin staining pattern, we evaluated the distribution of the clinicopathologic data and biomarkers (Phospho-Rb S249, N-cadherin, and β-catenin) expression (Tables 8 and 9) in each category. The distribution of the clinicopathologic data showed significant differences within the stage (*p*-value: <0.001), grade (*p*-value: <0.001), Gleason score (*p*-value: <0.001), and tumor size (*p*-value: <0.001) (Table 8). A detailed comparison of paired and unpaired analysis of the data presented in Table 8 is provided in Supplementary Table S4. Additionally, our findings revealed that patients with normal E-cadherin expression exhibited significantly higher levels of membranal β-catenin expression (*p*-value: <0.001) (Table 9). These suggest that E-cadherin and β-catenin might be potential biomarkers for PCa aggressiveness. Furthermore, we developed a logistic regression model to predict the likelihood of having an aggressive PCa based on E-cadherin staining patterns (N = 396). The model revealed that the combined expression of N-cadherin (OR: 1.27) and β-catenin (OR: 0.21) has the potential to identify patients with aberrant E-cadherin staining patterns (Table 10). The results suggest that lower β-catenin and higher N-cadherin expression are associated with a higher likelihood of having an aberrant E-cadherin expression. In addition, the model incorporated patients’ clinicopathologic data to confirm aggressive features. The results showed that age ≥ 80 years (OR: 6.98), grade 3 (OR: 12.86), stage 4 (OR: 10.57), and Gleason score ≥ 4 + 3 (OR: 7.66) had a strong influence on aggressive PCa outcomes. The model’s performance was calculated by measuring the pseudo-R^2^, obtaining a value of 0.4327, indicating that approximately 43.27% of the variability can be explained by the model, having a moderate ability to classify the patients by their E-cadherin staining patterns. These findings support the combined expression of E-cadherin, N-cadherin, and β-catenin as predictive indicators of aggressive PCa.

#### 3.4.3. Classification Tree

**Figure 6.**
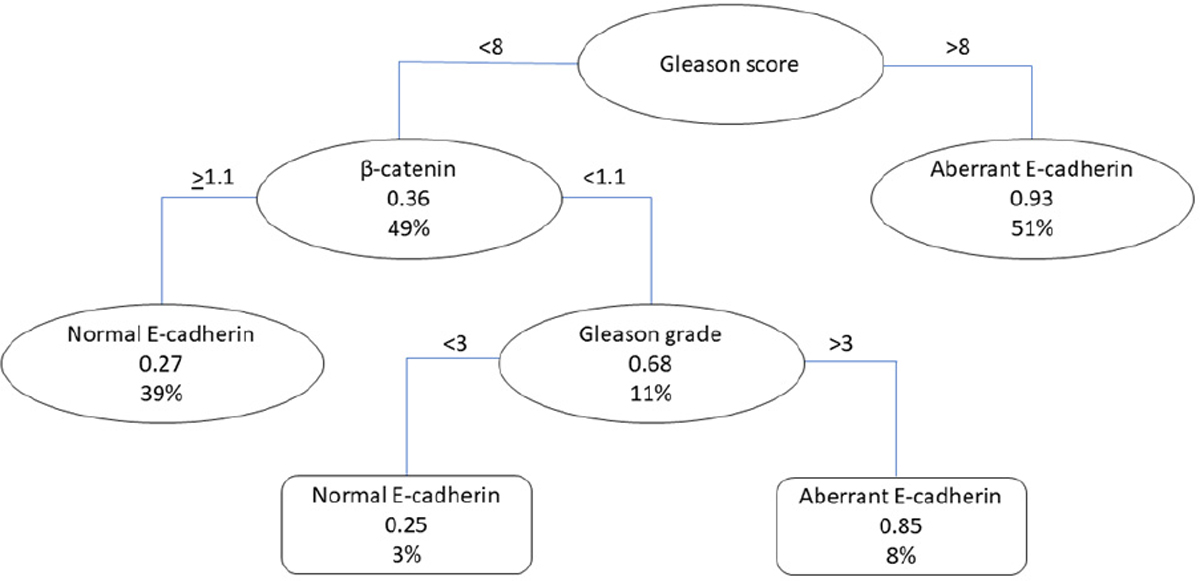
Classification tree for identifying PCa with aberrant E-cadherin staining patterns (N = 396).

The classification tree in Figure 6 shows the prediction of whether a patient has a normal or aberrant E-cadherin staining pattern, associated with tumor aggressiveness. The tree consists of five nodes and two leaves, and the variables selected by the model as important predictors were β-catenin, the Gleason score, and the Gleason grade. The primary split of the three was based on the Gleason score, where patients with scores > 8 had a high probability (93%) of having an aberrant E-cadherin staining pattern. Conversely, in patients with Gleason scores < 8, β-catenin expression levels were considered. There is a probability of a normal E-cadherin staining pattern of 0.27 (27%) if β-catenin expression levels are equal to or higher than 1.1, but if the β-catenin levels are lower than 1.1, further distinctions are made using the Gleason grade. For patients with Gleason grades < 3, the probability of having a normal E-cadherin staining pattern is 0.25 (25%), but if the Gleason grade is >3, the probability increases to 0.85 (85%). The model’s performance was evaluated using several metrics, including the AUC (training: 0.8499, testing: 0.8538), pseudo-R^2^ values (training: 0.2910, testing: 0.2192), sensitivity (0.8617), specificity (0.8246), the PPV (0.7297), the NPV (0.9156), and balanced accuracy (0.8431), indicating good discriminatory ability and overall performance, but also suggesting that there is still some variability that remains unexplained by the model (Table 6).

#### 3.4.4. Regression Tree

**Figure 7.**
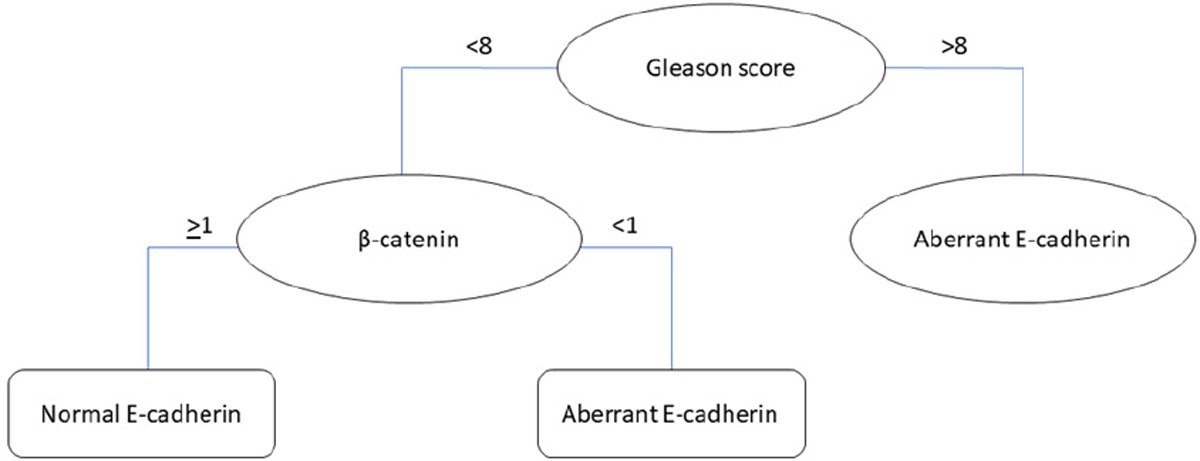
Regression tree for identifying PCa with aberrant E-cadherin staining patterns (N = 396).

The regression tree in Figure 7 predicts the likelihood of patients with a normal or aberrant E-cadherin staining pattern. This tree comprises three nodes and two leaves. The primary split in the model starts with the Gleason score, with values higher than 8 predicting an aberrant E-cadherin pattern. For patients with Gleason scores less than 8, the model further divided the patients based on β-catenin expression levels, with levels less than 1 indicating a higher likelihood of an aberrant pattern. Conversely, patients with β-catenin levels greater than 1 were predicted to have a normal E-cadherin staining pattern. The model’s effectiveness was evaluated by measuring R2 and RMSE (Table 7). An R2 value of 0.3845 indicates a moderate level of explanatory power, as approximately 38.45% of the variability in the E-cadherin staining pattern was explained by the model. In addition, an RMSE value of 1.1013 suggests a relatively high level of prediction error.

## 4. Discussion

Our study presents an innovative biomarker-based method for the early detection of aggressive PCa. We developed a novel approach by measuring the expression of key biomarkers (Phospho-Rb S249, N-cadherin, β-catenin, and E-cadherin) in PCa TMAs and applied logistic regression and CART models to identify patterns associated with aggressive PCa. These biomarkers have been correlated with clinicopathological data, indicating an association with the acquisition of aggressive PCa features. Notably, our findings suggest that the positive correlation between Phospho-Rb S249 and N-cadherin with clinicopathological data suggest a potential increase in aggressive tumor behavior. This is supported by the fact that Rb phosphorylation is associated with the loss of its tumor-suppressive capabilities, whereas N-cadherin, a mesenchymal marker, is linked to increased cell migration and invasion [31,32]. Additionally, the decreased expression of E-cadherin and β-catenin may be linked to the acquisition of aggressive tumor behavior, since they showed negative correlations with the clinicopathological data. It is well known that these biomarkers are crucial for maintaining cellular adhesion, and their loss has been associated with the development of EMT and tumor dissemination, which supports our findings [33]. While the selected biomarkers may not directly correlate with metastasis, it is crucial to remember that metastasis is a later event in aggressive PCa progression [34]. The goal of the evaluated biomarkers is to identify patients at risk before metastasis develops, allowing for earlier intervention and potentially improving patient outcomes.

This study stratified PCa patients based on Gleason scores and E-cadherin staining patterns, revealing insights that could be translated into clinical practice. A logistic regression model showed that the patients with Gleason scores ≥ 4 + 3 had a decreased membranal β-catenin expression, indicating a statistically significant relationship between β-catenin and the Gleason score. This suggests that β-catenin is an important biomarker for the early identification of patients with aggressive PCa. Additionally, the classification tree generated for detecting patients with Gleason scores ≥ 4 + 3 provides valuable insights into the role of β-catenin, tumor grade, and Gleason grade in predicting aggressive PCa. The high AUC, sensitivity, specificity, positive predictive value, and negative predictive value of the model suggest that it can make accurate predictions in a clinical setting. Thus, the model can potentially be a useful tool for the early identification of aggressive PCa, allowing clinicians to control disease progression, leading to better prognosis and quality of life for patients. However, the regression tree generated for detecting patients with Gleason scores ≥ 4 + 3 also showed robust and accurate outcomes. This model effectively identified the Gleason scores based on E-cadherin and β-catenin expression levels combined with the tumor grade and Gleason grade. However, clinicians should be aware that there may be occasional discrepancies in the predictions owing to the RMSE value. A common feature among all models developed for Gleason score classification was the inclusion of β-catenin as a determinant variable. As an important player in the Wnt signal transduction pathway, β-catenin’s accumulation in the cytoplasm and subsequent nuclear transport functions as a transcription factor, activating genes that drive uncontrolled cell proliferation in tumor cells [35,36]. Thus, the decreased membranal expression of β-catenin in patients with Gleason scores ≥ 4 + 3 may indicate its relocation to the cytoplasm or nucleus, a hallmark of aggressive cancer features.

This study also explored the differences in E-cadherin staining patterns, which are crucial for maintaining epithelial integrity. Through logistic regression, we found that N-cadherin and β-catenin expression could potentially classify patients by their E-cadherin staining patterns. The model’s clinicopathological data, including age, grade, stage, and Gleason score, significantly contributed to the detection of patients with aberrant E-cadherin staining patterns. Although the model has moderate explanatory power, it requires further refinement to account for unexplained variability and improve accuracy. In contrast, the classification tree developed for the identification of patients with aberrant E-cadherin staining patterns has been shown to perform well in terms of discrimination. However, it possesses a moderate level of fit, with significant unexplained variance, suggesting that additional predictors or a more complex model might improve its explanatory power. In addition, the regression tree revealed that Gleason scores and β-catenin expression levels play a significant role in determining the likelihood of aberrant E-cadherin staining. Higher Gleason scores and low β-catenin levels are associated with a greater probability of aberrant E-cadherin staining patterns, which is linked to a higher risk of aggressive PCa. However, the model’s performance indicates that even with important predictors such as the Gleason score and β-catenin levels, it may benefit from additional variables or complex interactions to improve its explanatory power. Its predictive accuracy suggests that the model’s predictions are relatively inaccurate; therefore, enhancements may be needed to improve its clinical use.

The comparison of logistic regressions, classification trees, and regression trees for identifying aggressive PCa reveals that models based on Gleason scores are the most effective. The E-cadherin-based models show moderate performance but with lower explanatory power. The logistic regression model for Gleason score classification showed a relevant negative association with β-catenin but incorporated significant variance. On the other hand, the regression tree showed good performance, with a substantial R^2^ but a higher RMSE, suggesting lower precision in predictions. In contrast, the decision tree achieves the highest accuracy and variance explanation, with high AUC values and substantial pseudo-R^2^ values. The classification tree also exhibits high sensitivity, specificity, and balanced accuracy, reinforcing its reliability. Even though the classification tree for Gleason score classification stands out, exhibiting robust and reliable predictions, the regression tree for Gleason score classification also shows good performance but with lower precision in predictions. Both the regression tree and decision tree for Gleason score classification incorporate the same variables, including β-catenin, grade, and Gleason grade, except for E-cadherin, which is only included in the regression tree. Thus, the combined expression of β-catenin with grade, Gleason grade, and Gleason score shows promise for improving the early identification of aggressive PCa and informing personalized treatment strategies to control tumor growth, prevent metastasis, and manage symptoms. Furthermore, a prospective study is necessary to validate the clinical utility of the classification tree in PCa risk assessment. This approach will enable the gathering of additional patient data that are not provided in TMA reports, such as medical records, ethnicity, and potential MRI results, to improve the early identification of aggressive PCa.

## 5. Conclusions

The classification tree’s outstanding performance demonstrates its ability to accurately differentiate between Gleason scores, providing a reliable tool for clinical decision-making and enhancing patient stratification by integrating β-catenin expression levels and clinicopathological variables. The model’s high accuracy strongly supports its potential to predict aggressive PCa, enabling early identification of cases that may benefit from earlier treatment intervention before metastasis occurs. Future studies should investigate the incorporation of additional biomarkers and validate the model using independent datasets to ensure its robustness and generalizability.

## Supplementary Material

Sup Tables 1-4

**Supplementary Materials:** The following supporting information can be downloaded at: https://www.mdpi.com/article/10.3390/stats7030053/s1, Table S1: Logistic regression models for individual biomarkers; Table S2: Logistic regression models for two biomarkers combinations; Table S3: Logistic regression models for three biomarkers combinations; Table S4: Statistical comparison of unpaired and paired tests for the clinicopathologic data within the membrane and aberrant E-cadherin expression.

## Figures and Tables

**Table 1.** Correlation of the patient’s clinicopathologic data with the expression of Phospho-Rb S249 (N = 403).

Variables	*p*-Value	r

Tumor size	<0.0001	0.4711
Lymph node invasion	0.3902	0.06407
Metastasis	0.5106	0.04907
Grade	0.0910	0.1320
Stage	<0.0001	0.4405
Gleason grade	0.3703	0.06874
Gleason score	0.3982	0.06463

**Table 2.** Correlation of N-cadherin, β-catenin, and E-cadherin with the patient’s clinicopathologic data (N = 403).

N-Cadherin	*p*-Value	r

Tumor size	<0.0001	0.3782
Lymph nodes invasion	0.2540	0.08498
Metastasis	0.0663	0.1364
Grade	0.3585	0.07193
Stage	<0.0001	0.3522
Gleason grade	0.0313	0.1643
Gleason score	0.1016	0.1245
β-catenin	*p*-value	r
Tumor size	0.0119	−0.1861
Lymph node invasion	0.6122	−0.03782
Metastasis	0.3912	0.06394
Grade	0.0004	−0.2716
Stage	0.0051	−0.2069
Gleason grade	0.0010	−0.2495
Gleason score	0.0002	−0.2810
E-cadherin	*p*-value	r
Tumor size	0.0002	−0.2746
Lymph node invasion	0.4974	−0.05061
Metastasis	0.9504	0.004647
Grade	<0.0001	−0.2984
Stage	<0.0001	−0.3567
Gleason grade	0.0020	−0.2338
Gleason score	0.0008	−0.2530

**Table 3.** Clinicopathologic characteristics of PCa patients by their Gleason scores. The table includes the number of patients (N) and the percentage contribution in each category, as well as the results of statistical analysis using an unpaired *t*-test.

Variables	Overall (N = 396)	Gleason Score ≤ 3 + 4 (N = 195)	Gleason Score ≥ 4 + 3 (N = 201)	*p*-Value

Tumor size (N = 396)				0.0002

1	4 (1.01)	4 (2.05)	0	
2	233 (58.84)	129 (66.15)	104 (51.74)	
3	136 (34.34)	55 (28.21)	81 (40.30)	
4	23 (5.81)	7 (3.59)	16 (7.96)	

Lymph node invasion (N = 396)				0.1428

0	366 (92.42)	176 (90.26)	190 (94.53)	
1	28 (7.07)	18 (9.23)	10 (4.98)	
2	2 (0.51)	1 (0.51)	1 (0.50)	

Stage (N = 396)				0.0018

1	27 (6.82)	24 (12.30)	3 (1.49)	
2	214 (54.04)	107 (54.87)	107 (54.87)	
3	110 (27.78)	43 (22.05)	67 (33.33)	
4	45(11.36)	21 (10.77)	24 (11.94)	

Metastasis (N = 396)				0.0089

No	386 (97.47)	186 (95.38)	200 (99.50)	
Yes	10 (2.53)	9 (4.62)	1 (0.50)	

Grade (N = 394)				0.0000

1	33 (8.38)	33 (16.92)	0	
2	194 (49.24)	147 (75.38)	47 (23.38)	
3	167 (42.39)	10 (5.13)	157 (78.11)	

Gleason grade (N = 396)				0.0000

1	18 (4.55)	18 (9.23)	0	
2	48 (12.12)	48 (24.62)	0	
3	84 (21.21)	81 (41.54)	3 (1.49)	
4	131 (33.08)	48 (24.62)	83 (41.29)	
5	115 (29.04)	0	115 (57.21)	

Gleason score (N = 396)				-

1	0	0	0	
2	3 (0.75)	3 (1.54)	0	
3	8 (2.02)	8 (4.10)	0	
4	12 (3.03)	12 (6.15)	0	
5	23 (5.80)	23 (11.79)	0	
6	46 (11.62)	46 (23.59)	0	
7	103 (26.01)	103 (52.82)	0	
8	67 (16.92)	0	67 (33.33)	
9	66 (16.67)	0	66 (32.84)	
10	68 (188.89)	0	68 (33.83)	

**Table 4.** Expression of the biomarker’s Phospho-Rb S249, N-cadherin, β-catenin, and E-cadherin in PCa patients stratified by their Gleason scores. The table includes the mean, standard deviation (sd), median, and percentiles (p) for each biomarker in each category.

Variables	Overall (N = 396)	Gleason Score ≤ 3 + 4 (N = 195)	Gleason Score ≥ 4 + 3 (N = 201)	*p*-Value

Age				

Mean (sd)	69.00 (8.79)	68.36 (6.88)	69.63 (10.29)	0.1532
Median (p25, p75)	70 (64, 75)	69 (64, 73)	70 (64, 76)	

Phospho-Rb S249				

Mean (sd)	2.72 (0.70)	2.79 (0.69)	2.64 (0.71)	0.0304
Median (p25, p75)	2.84 (2.33, 3.21)	2.93 (2.33, 3.31)	2.79 (2.33, 3.12)	

N-Cadherin				

Mean (sd)	1.85 (0.58)	1.90 (0.61)	1.81 (0.55)	0.1094
Median (p25, p75)	1.88(1.29, 2.31)	1.92 (1.29, 2.38)	1.88 (1.33, 2.21)	

β-Catenin				

Mean (sd)	1.59 (0.62)	1.70 (0.64)	1.48 (0.57)	0.0003
Median (p25, p75)	1.38 (1, 2)	1.92 (1.13, 2.25)	1.21 (1, 1.83)	

E-Cadherin				

Mean (sd)	2.20 (0.64)	2.26 (0.62)	2.15 (0.66)	0.0826
Median (p25, p75)	2.33 (1.67, 2.72)	2.38 (1.75,2.75)	2.21 (1.58, 2.71)	

**Table 5.** Logistic regression model for identifying PCa with Gleason scores ≥ 4 + 3 (N = 396).

Variable	OR (CI)
β-catenin	0.55 (0.39–0.77)

**Table 6.** Performance metrics for Gleason score and E-cadherin staining pattern classification models.

Variables	Gleason Score Classification	E-Cadherin Staining Pattern Classification
Training AUC	0.9572	0.8499
Testing AUC	0.9621	0.8538
Pseudo-R^2^ training	0.7401	0.291
Pseudo-R^2^ testing	0.6294	0.2192
Sensitivity	0.9655	0.8617
Specificity	0.9133	0.8246
PPV	0.896	0.7297
NPV	0.9716	0.9156
Prevalence	0.4361	0.3547
Detection rate	0.4211	0.3057
Detecti on prevalence	0.4699	0.4189
Balanced accuracy	0.9394	0.8431

**Table 7.** Performance metrics for Gleason score and E-cadherin staining pattern regression models.

Variable	Gleason Score Classification	E-Cadherin Staining Pattern Classification
R^2^	0.6961	0.3844
RMSE	1.0247	1.1013

**Table 8.** Clinicopathologic data comparison within the membrane and aberrant E-cadherin expression. The table includes the number of patients (N) and percentage of contribution in each category. After separating the patients by their E-cadherin staining patterns, a normality test revealed that the data are normally distributed, as confirmed by a Shapiro–Wilk test. Thus, an unpaired *t*-test was selected for this analysis.

Variables	Membrane (N = 143)	Aberrant (N = 303)	*p*-Value

Stage (N = 407)	N = 139	N = 268	<0.001

1	22	6	
2	79	138	
3	24	88	
4	12	34	
Median	2	2	

Grade (N = 392)	N = 134	N = 258	<0.001

1	29	4	
2	91	103	
3	12	148	
4	0	0	
5	0	0	
Median	2	3	

Gleason score (N = 439)	N = 141	N = 298	<0.001

1	0	0	
2	3	0	
3	8	0	
4	9	3	
5	20	3	
6	33	17	
7	53	53	
8	5	80	
9	4	71	
10	6	71	
Median	6	8	

Tumor size (T) (N = 403)	N = 137	N = 266	<0.001

0	0	0	
1	2	2	
2	98	139	
3	33	106	
4	4	19	
Median	2	2	

Lymph node invasion (N) (N = 403)	N = 137	N = 266	0.580

0	128	245	
1	9	19	
2	0	2	
Median	0	0	

Metastasis (M) (N = 403)	N = 137	N = 266	0.279

No	132	261	
Yes	5	5	
2	0	0	
Median	0	0	

**Table 9.** Expression of Phospho-Rb S249, N-cadherin, and β-catenin, stratified by E-cadherin staining patterns. The table displays the mean, standard deviation, median, and percentiles (e.g., 25th and 75th percentiles) for each biomarker within each E-cadherin staining pattern. All *p*-values were analyzed following an unpaired *t*-test unless otherwise stated.

Variable	Overall (N = 403)	Membrane (N = 137)	Aberrant (N = 266)	*p*-Value

Phospho-Rb S249				

Mean (sd)	2.72 (0.70)	2.75 (0.71)	2.71 (0.70)	0.500
Median (p25, p75)	2.79 (2.33,3.21)	2.88 (2.33, 3.25)	2.75 (2.38, 3.17)	

N-Cadherin				

Mean (sd)	185 (0.58)	1.92 (0.62)	1.81 (0.56)	0.062
Median (p25, p75)	1.83 (1.29, 2.29)	1.92 (1.29, 2.38)	1.74 (1.29, 2.21)	

E-Cadherin				-

Mean (sd)	2.19 (0.64)	2.41(0.52)	2.08 (0.67)	
Median (p25, p75)	2.21 (1.67, 2.71)	2.42 (2.08, 2.79)	2.04 (1.50, 2.67)	

β-Catenin				<0.001 ^1^

Mean (sd)	1.59 (0.61)	1.87 (0.67)	1.44 (0.53)	
Median (p25, p75)	1.38 (1, 2)	1.71 (1.29, 2.46)	1.23 (1.00, 1.71)	

1 Wilcoxon test.

**Table 10.** Logistic regression model for the identification of aggressive PCa, stratified by E-cadherin staining patterns. The table shows the odds ratios (ORs) and confidence intervals (CIs) for each variable included in the model (N = 396).

Variables	OR (CI)

β-catenin	0.21 (0.12–0.38)

N-cadherin	1.27 (0.73–2.22)

Age (≥ 80 years)	6.98 (1.23–39.60)

Grade	

1	-
2	4.25 (1.27–14.19)
3	12.86 (2.73–60.61)

Stage	

1	-
2	4.13 (0.82–20.79)
3	5.72 (1.05–31.22)
4	10.57 (1.69–66.00)

Gleason score (≥4 + 3)	7.66 (2.80–20.94)

## Data Availability

The raw data supporting the conclusions of this article will be made available by the authors on request.
