# Supplementary material for: Integrating Proteomic Analysis and Machine Learning to Predict Prostate Cancer Aggressiveness
Source: Stats (Basel). Author manuscript; Available in PMC 2025 Oct 4. (PMC12494234; doi:10.3390/stats7030053)
Supplement: Sup Tables 1-4 [file NIHMS2111425-supplement-Sup_Tables_1-4.pdf]

Supplementary Table S1. Logistic regression models for individual biomarkers

**Variable**	**OR (CI)**	***p*-value**
Phospho-Rb S249	0.8373 (0.62-1.13)	0.246
N-cadherin	0.8970 (0.65-1.23)	0.503
E-cadherin	0.7345 (0.55-0.97)	0.034
β-catenin	0.4780 (0.35-0.65)	<0.000

Supplementary Table S2. Logistic regression models for two biomarkers combinations

**Variable**	**OR (CI)**	***p*-value**
**Combination 1**		
Phospho-Rb S249	0.7401(0.55-0.98)	0.042
N-cadherin	0.9532(0.68-1.31)	0.772
**Combination 2**		
Phospho-Rb S249	0.7459(0.56-0.99)	0.045
E-cadherin	0.8610(0.63-1.16)	0.334
**Combination 3**		
Phospho-Rb S249	0.8704(0.64-1.17)	0.364
β-catenin	0.4990 (0.36-0.68)	<0.000
**Combination 4**		
N-cadherin	0.9613(0.67-1.36)	0.824
E-cadherin	0.8498(0.61-1.17)	0.33
**Combination 5**		
N-cadherin	1.1495(0.81-1.62)	0.426
β-catenin	0.4594(0.33-0.63)	<0.000
**Combination 6**		
E-cadherin	1.3792(0.95-1.99)	0.087
β-catenin	0.4003(0.27-0.58)	<0.000

Supplementary Table S3. Logistic regression models for three biomarkers combinations

**Variable**	**OR (CI)**	***p*-value**
**Combination 1**		
Phospho-Rb S249	0.8568(0.63-1.15)	0.316
β-catenin	0.4791(0.34-0.66)	<0.000
N-cadherin	1.1713(0.82-1.65)	0.37
**Combination 2**		

Phospho-Rb S249	0.8779(0.64-1.18)	0.397
β-catenin	0.4197(0.28-0.61)	<0.000
E-cadherin	1.3609(0.94-1.97)	0.103
**Combination 3**		
Phospho-Rb S249	0.7443(0.55-0.99)	0.046
N-cadherin	1.0176(0.71-1.44)	0.922
E-cadherin	0.8555(0.61-1.19)	0.354
**Combination 4**		
N-cadherin	1.0569(0.73-1.51)	0.763
E-cadherin	1.3552(0.92-1.99)	0.123
β-catenin	0.3979(0.27-0.57)	<0.000

Supplementary Table S4. Statistical comparison of unpaired and paired tests for the clinicopathologic data within the membrane and aberrant E-cadherin expression. This table presents a comparative analysis of the results obtained from unpaired and paired t-tests for the clinicopathologic data associated with membrane and aberrant E-cadherin expression. Prior to conducting the unpaired t-test, a Shapiro-Wilk normality test was performed to assess the distribution of the data and ensure normality. In contrast, the paired t-test was conducted following a Wilcoxon rank-sum test to account for non-parametric assumptions.

**Variables**	**Membrane (N=143)**	**Aberrant (N=303)**	**Unpaired t-test *p*-value**	**Paired t-test *p*-value**
Stage (N=407)	N=139	N=268	<0.001	<0.0001
1	22	6		
2	79	138		
3	24	88		
4	12	34		
Median	2	2		
Grade (N=392)	N=134	N=258	<0.001	<0.0001
1	29	4		
2	91	103		
3	12	148		
4	0	0		
5	0	0		
Median	2	3		
Gleason score (N=439)	N=141	N=298	<0.001	<0.0001
1	0	0		
2	3	0		
3	8	0		

4	9	3		
5	20	3		
6	33	17		
7	53	53		
8	5	80		
9	4	71		
10	6	71		
Median	6	8		
Tumor size (T) (N=403)	N=137	N=266	<0.001	<0.0001
0	0	0		
1	2	2		
2	98	139		
3	33	106		
4	4	19		
Median	2	2		
Lymph nodes invasion (N) (N=403)	N=137	N=266	0.580	0.2114
0	128	245		
1	9	19		
2	0	2		
Median	0	0		
Metastasis (M) (N=403)	N=137	N=266	0.279	>0.9999
No	132	261		
Yes	5	5		
2	0	0		
Median	0	0		
